# Ecological strategies and extinction risk in butterflies and macro-moths of Great Britain and Ireland: A trait similarity network approach

**DOI:** 10.1371/journal.pone.0348053

**Published:** 2026-05-13

**Authors:** Lacey Boltz, Kaitlyn Kelble, Janice Tsogbe, John Matta

**Affiliations:** Department of Computer Science, Southern Illinois University Edwardsville, Edwardsville, Illinois, United States of America; University of Padova: Universita degli Studi di Padova, ITALY

## Abstract

Trait-based analyses of extinction risk often examine individual traits independently, which can obscure how combinations of traits define broader ecological strategies. Here, we apply a network-based representation of trait similarity to analyze how extinction risk is distributed across combinations of ecological traits, extending an approach previously applied to seabirds. Using a curated species-level trait dataset for butterflies and macro-moths (order Lepidoptera), we constructed a species similarity network for 968 species and identified nine ecological communities using unsupervised community detection. The resulting communities were structured primarily by phenology and life-cycle characteristics. Although conservation status was not used in clustering, threatened species were unevenly distributed across communities, with the multivoltine, externally developing strategy group containing nearly six times the expected number of threatened species relative to background prevalence in both Great Britain and Ireland. These results show that extinction risk in Lepidoptera is structured by integrated ecological strategies, with patterns of vulnerability emerging from multivariate trait configurations. Trait similarity networks therefore provide a scalable approach for identifying strategy-level patterns of conservation vulnerability beyond single-species assessments, particularly in data-limited taxa such as insects where long-term population monitoring is often incomplete.

## Introduction

Understanding why extinction risk is concentrated in some species but not others remains a central challenge in conservation biology. A large body of comparative research has linked individual traits, such as body size [1], reproductive rate [2], ability to move between habitats (dispersal) [3], and habitat specialization [4], to increased vulnerability, yet relationships between these traits will manifest differently across taxa and regions. Increasingly, it is recognized that extinction risk arises from interacting combinations of traits that jointly shape how species persist in their environments, with single attributes alone providing only limited explanation of overall vulnerability. This pattern is demonstrated clearly in butterflies and macro-moths (order Lepidoptera), where suites of life-history traits linked to host-plant strategies are associated with differences in rarity and conservation status [5].

Trait-based approaches have therefore become a cornerstone of conservation assessments [6]. By summarizing how species acquire resources, reproduce, and survive, functional traits provide a bridge between species biology and population-level outcomes [7]. However, many trait-based analyses rely on additive statistical models that treat traits as independent predictors of extinction risk [8]. Such approaches can obscure interactions among traits and may fail to capture higher-order structure arising from interacting trait combinations.

This limitation is particularly important in insect conservation, where long-term population monitoring data are often incomplete or unavailable for many species. In such systems, trait-based approaches provide an essential alternative framework for identifying species and ecological strategies likely to be vulnerable, allowing conservation risk to be assessed even when direct demographic information is limited. As a result, trait-based analyses play an especially important role in improving extinction-risk inference across diverse and understudied insect taxa.

Network-based representations of trait similarity offer an alternative framework for addressing these limitations. In this context, trait-based network analysis represents species as nodes connected by edges that quantify their similarity across multiple ecological traits, allowing groups of species with similar combinations of life-history characteristics to be identified as communities within trait space. Unlike additive trait-based models that evaluate predictors independently, this representation captures interactions among traits and allows vulnerability to be examined at the level of integrated ecological strategies. By representing species as nodes connected by edges reflecting overall similarity, trait similarity networks preserve structure arising from combinations of attributes through simultaneous comparison across multiple traits, capturing multivariate ecological structure in a single representation [9]. The communities can be interpreted as integrated ecological profiles that may share common sensitivities to environmental change [10]. While ecological networks have been widely used to study species interactions, their application to identifying non-random patterns of conservation risk based on trait similarity remains relatively underexplored, even though biodiversity loss is increasingly recognized to reshape communities by disproportionately affecting species with particular combinations of life-history traits and ecological strategies [11].

In prior work we applied a network-based trait similarity approach to seabirds, demonstrating that extinction vulnerability was strongly clustered within a distinct phenotypic guild and was not evenly distributed across species [12]. Using a weighted phenotypic similarity network, that study identified four coherent trait-based communities, one of which contained nearly half of all threatened species. Importantly, elevated risk emerged from combinations of traits related to body size, foraging and feeding mode, locomotion, and migration, not from any single defining characteristic alone. These results illustrated the potential of trait similarity networks to reveal combination-based vulnerability that is not apparent from additive trait analyses. Based on prior seabird results, we hypothesized that extinction risk would cluster within specific regions of Lepidoptera trait space instead of being randomly distributed across species.

Because trait similarity networks group species according to phenotypic similarity and not shared ancestry (phylogeny), the resulting communities do not necessarily correspond to phylogenetic clades. Instead, they represent ecological strategy groupings that may arise through either shared evolutionary history or convergent life-history structure. Evaluating the relative contributions of phylogeny and ecological similarity represents an important direction for future work.

The seabird analysis highlighted several opportunities for methodological extension. The trait set was limited in scope and resolution, network construction relied on a simple similarity formulation without explicit feature selection, and robustness to alternative parameter choices was not systematically assessed. Together, these considerations motivate the development of a more general and scalable framework for applying trait-based network analysis to conservation questions.

Here, we extend this approach to Lepidoptera, a taxonomic group of major conservation concern and broad ecological importance. Lepidoptera exhibit pronounced variation in life-history strategies such as reproductive and overwintering modes [13], strong phenological diversity including cyclic phenomena such as egg-laying and migration timing [14], a wide range of developmental environments spanning habitats and climate zones [15], and diverse host associations reflecting varying degrees of specialization [16]. As a result, Lepidoptera have been widely used as indicators of environmental change [17].

There is no a priori reason to expect that the same traits should be associated with extinction risk across taxonomic groups, because vulnerability reflects interactions between intrinsic life-history characteristics and the external threats acting within particular ecological systems. Traits linked to increased risk in one taxonomic group may therefore differ substantially from those associated with vulnerability in another [18]. Instead, extending trait similarity network approaches across taxa provides an opportunity to test whether extinction risk consistently concentrates within multivariate ecological strategy groups, even when the traits defining those strategies differ.

Identifying trait-space structure is useful because species that share ecological strategies often respond similarly to environmental change, allowing conservation monitoring to be organized around strategy groups instead of individual species assessments alone. This perspective is particularly important for insects, where long-term population monitoring data remain unavailable for many species and conservation assessments must often rely on indirect indicators of vulnerability. Because ecological trait information is available for substantially larger numbers of species than demographic trend data, trait-based approaches provide an especially valuable framework for identifying shared risk structure across data-limited taxa such as Lepidoptera. Lepidoptera therefore provide an ideal system for evaluating whether the vulnerability clustering observed previously in seabirds also extends to taxa structured primarily by phenological and developmental traits, with less emphasis on movement and foraging ecology. Compared with seabirds, Lepidoptera trait datasets are larger, more heterogeneous, and dominated by categorical life-history descriptors, posing additional challenges for network construction and interpretation.

Previous work has identified several ecological correlates of extinction risk in Lepidoptera. For example, Koh et al. [19] emphasized the importance of ecological specialization and host use in shaping vulnerability. Franzen and Johannesson [20] examined how distribution patterns and species characteristics predict local extinction risk across butterflies and moths. Nylin and Bergström [21] highlighted links between life-history characteristics, such as host-plant associations and developmental strategy, and broader vulnerability patterns. Franke et al. [22] provided further evidence, in European butterflies and odonates, that extinction risk is associated with ecological traits including specialization, dispersal-related characteristics, and habitat use. Together, these studies have substantially advanced understanding of vulnerability in Lepidoptera, but they have largely examined traits individually or within additive statistical frameworks, without asking whether species sort into broader multivariate strategy groups defined by recurring trait combinations. The present study addresses this gap by applying a trait similarity network framework to examine whether Lepidoptera form ecological communities in trait space and whether conservation risk is disproportionately structured across these strategy-level groupings.

In this study, we construct a species similarity network for Lepidoptera based on a binary trait incidence matrix with explicit biological labeling of trait values, an approach commonly used in functional trait analyses to represent species–trait relationships [6,7]. We introduce a feature selection pipeline designed to reduce redundancy while preserving ecologically meaningful structure, following recent work emphasizing stability-aware selection in high-dimensional trait representations [23]. Network construction parameters are evaluated using bootstrap-based robustness analyses to assess the consistency of detected structure across resampled datasets [24]. Ecological communities are then identified using unsupervised community detection methods widely applied in ecological network analysis, including modularity-based approaches such as the Leiden algorithm [25,26].

By applying this expanded framework, we address two primary questions. First, do Lepidoptera form coherent ecological communities in trait space that reflect multidimensional life-history strategies? Second, is conservation risk disproportionately concentrated within particular trait-defined communities, analogous to patterns observed previously in seabirds? By addressing these questions, we demonstrate how trait similarity networks can serve as a general tool for identifying ecological strategies associated with elevated vulnerability and for informing conservation monitoring beyond single-species assessments. Based on prior results in seabirds, we specifically test whether extinction risk clusters within particular regions of Lepidoptera trait space instead of being spread randomly across species. The goal of this analysis is not to predict the conservation status of individual species, but to identify regions of ecological strategy space in which extinction risk is disproportionately concentrated.

## Related work

### Trait-based approaches to extinction risk

Trait-based approaches to extinction risk are widely used, but cross-taxonomic studies have shown that the same trait can be associated with vulnerability in one group and not in another, making it difficult to apply findings across groups [27]. Subsequent studies have further emphasized that the predictive performance of trait-based extinction models is often modest and sensitive to both modeling assumptions and environmental context [28]. Together, these findings suggest that trait-based approaches capture important but incomplete dimensions of extinction risk, motivating continued methodological development. Recent work has further shown that conservation-risk assessment in butterflies can be strengthened by integrating heterogeneous evidence sources, including demographic, climatic, and trait-based information, particularly when data availability varies substantially across species [29].

Many trait-based extinction studies have relied on statistical models that evaluate the effects of individual traits separately. Early comparative analyses using this approach identified clear associations between specific traits and extinction risk, but also revealed substantial unexplained variation and sensitivity to analytical choices [30]. Subsequent studies incorporating demographic data and improved predictors have shown that these limitations persist, with predictive performance often constrained by correlations among traits and by assumptions that traits act independently and have simple, linear relationships with extinction risk, conditions that are rarely met in ecological systems with many interacting traits [8].

Ecological traits, however, are frequently correlated and interact to define composite survival strategies. Empirical studies examining trait interactions have shown that vulnerability often emerges from combinations of traits rather than from any single attribute alone, and that additive models may obscure higher-order structure in ecological strategies [31]. More comprehensive reviews of trait-based extinction studies have further emphasized that ignoring such interactions can lead to underestimation of the importance of integrated ecological strategies for long-term survival and stability [32]. Together, these findings have motivated interest in analytical frameworks that more directly capture multivariate trait structure.

### Network-based and multivariate approaches to ecological vulnerability

Network-based approaches provide a general framework for representing complex relationships among species without reducing them to independent variables. In ecology, network representations have been used not only to study species interactions such as food webs, but also to describe similarity relationships among species, patterns of trait co-occurrence, and other forms of functional and structural organization [33]. Analyses of ecological network representations have shown that networks can capture complex connections between many different traits that are difficult to express using traditional statistical models [34]. Related frameworks have further demonstrated how multiple ecological dimensions, including functional traits, can be incorporated within a single network representation [35]. In the context of trait analysis, species similarity networks offer a way to represent overall ecological resemblance among species without assuming the data follow a specific mathematical pattern.

Studies on ecological networks have shown that sensitivity to disturbance is often unevenly distributed across network structure. For example, analyses of interaction networks have demonstrated that species occupying particular positions or modules can be disproportionately associated with reduced stability or heightened sensitivity to species loss [36]. Other analyses have emphasized how the division of ecological networks into modules, or groups of more strongly connected species, influences how environmental change propagates through the system [37]. However, this body of work has focused primarily on interaction networks and network robustness, as opposed to networks derived from multivariate species trait similarity.

Building on these ideas, our previous work applied a trait-based species similarity network to seabirds, demonstrating that extinction risk was strongly clustered within a distinct phenotypic community, with no evidence of an even distribution across species [12]. That study provided a proof of concept for using trait similarity networks to identify groups of species facing similar threats arising from combinations of traits related to feeding, locomotion, and life-history strategy. However, the approach was applied to a relatively small dataset with limited trait resolution, and network construction parameters were fixed without systematic robustness assessment.

The present study extends this line of work by applying a trait-based network approach to Lepidoptera, a taxonomic group with diverse life-history strategies. By moving beyond a single taxon, we examine whether patterns of clustered conservation risk identified using trait similarity networks generalize across groups with very different ecological characteristics. The goal in doing this is to provide a broadly applicable framework for identifying ecological strategies associated with extinction risk.

## Methods

### Trait data

Trait data for Lepidoptera of Great Britain and Ireland were obtained from the NERC Environmental Information Data Centre [38]. The dataset provides species-level information on life-history, phenology, developmental environment, and host associations.

### Trait encoding and incidence matrix construction

**Fig 1:**
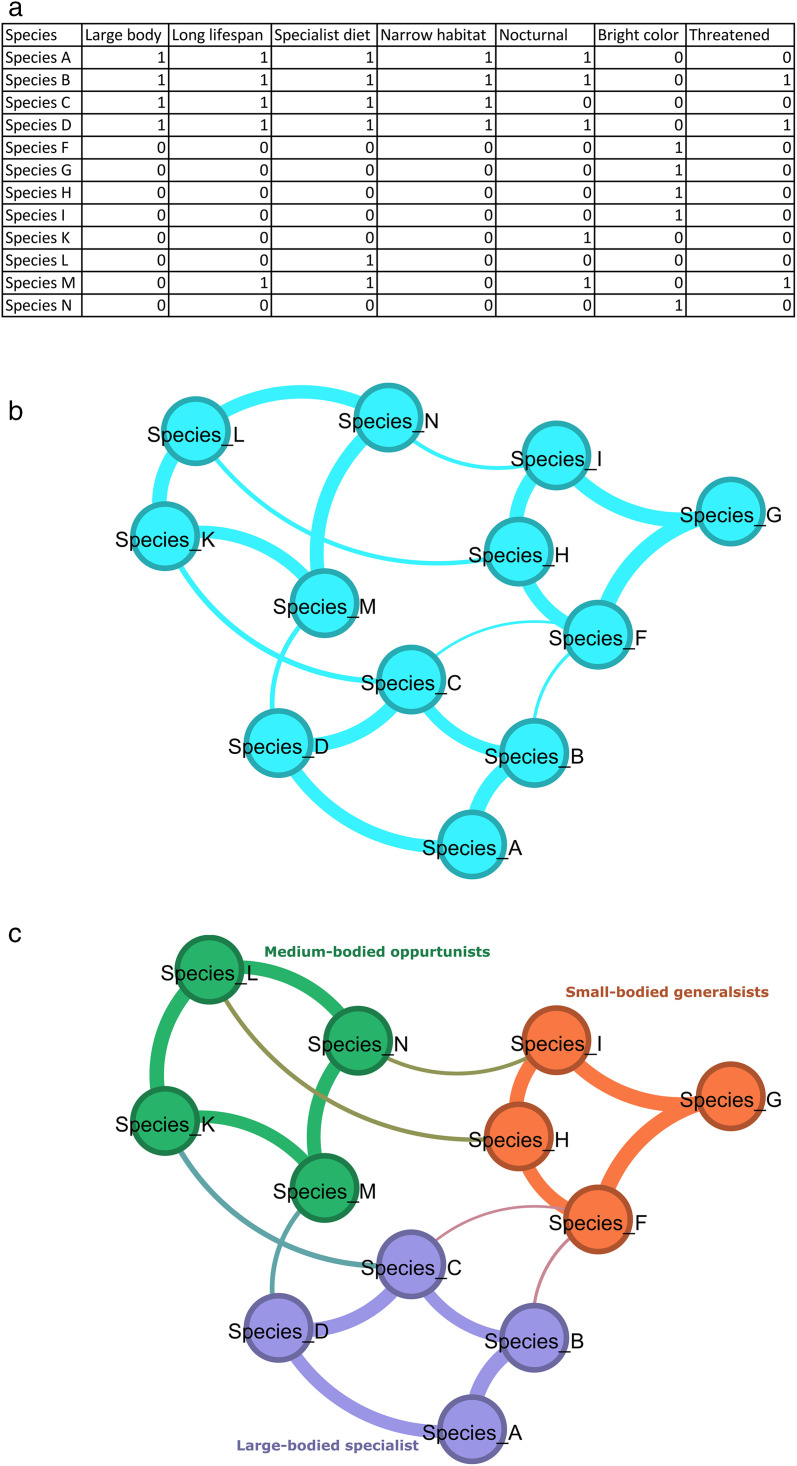
Construction of a trait similarity network from binary trait data. (a) Trait incidence matrix for 12 example species across seven binary traits. A value of 1 indicates trait presence and 0 indicates trait absence. (b) Trait similarity network constructed from the incidence matrix in panel (a). Each circle (node) represents a species, and each line (edge) connecting two species indicates that those species share a similar combination of traits, for example, both being large-bodied, nocturnal, and specialist feeders. Only species pairs with sufficiently similar trait profiles are connected, meaning species that share more traits in common are more likely to be linked. The thickness of each line reflects how similar the two connected species are, with thicker lines indicating a greater number of shared traits. (c) The same trait similarity network with nodes colored by ecological strategy groups: large-bodied specialists, small-bodied generalists, and medium-bodied opportunists. Clusters of densely connected nodes correspond to species sharing recurring combinations of life-history traits, representing distinct ecological strategies occupying similar regions of multidimensional trait space.

We constructed a species–trait incidence matrix in which rows correspond to Lepidoptera species and columns correspond to explicitly defined ecological trait-values. This matrix served as the basis for all subsequent network analyses. Trait-values were included only if they represented biologically meaningful ecological states, were defined consistently across species, and occurred with sufficient prevalence to support comparative analysis. Non-ecological metadata, database flags, and unresolved missing trait entries were not encoded as trait-values. This filtering applied to variables and trait encodings rather than to species. Species were excluded only from the post hoc conservation-status analyses when Red List status was classified as Data Deficient, Not Evaluated, or Regionally Extinct. The resulting incidence matrix provides the basis for constructing a species similarity network in which nodes represent species and edges represent similarity in encoded ecological trait profiles. The workflow for this process is illustrated in Fig 1.

All categorical traits were encoded using binary encoding, with each column representing a single trait-value. Column names were fully self-describing (e.g., *pupal_habit_soil*, *flight_season_early_spring*), allowing each trait-value to be interpreted independently without reference to the raw dataset. Encoding decisions were governed by an explicit trait dictionary that mapped raw data values to inclusion status and column labels, ensuring consistent trait definitions across species and reproducible construction of the incidence matrix.

Phenological and developmental timing information was encoded as binary indicators of presence during defined monthly or seasonal intervals, following the source data documentation. Where relevant, confirmed and uncertain states were retained as separate trait-values to preserve biologically meaningful distinctions. Pupal habit traits were encoded as mutually exclusive binary indicators representing primary pupation environments.

Continuous variables and derived summary metrics were not included in the incidence matrix. A summary of excluded continuous variables and the rationale for their omission is provided in Table 1. Many key Lepidoptera life-history characteristics, including voltinism, overwintering stage, and developmental environment, are more appropriately represented as discrete ecological states and were therefore encoded as categorical variables for network construction. The resulting incidence matrix contains binary indicators of trait presence or absence for each species, enabling transparent computation of species similarity and direct ecological interpretation of the resulting network communities.

**Table 1:** Continuous variables excluded prior to construction of the species–trait incidence matrix. Continuous morphological measurements, monitoring-derived distribution and trend metrics, phenological shift statistics, and hostplant Ellenberg indicator values were excluded because species similarity in this study was defined using categorical ecological trait states representing life-history strategies. Variables reflecting monitoring coverage, temporal change, morphological magnitude, or environmental proxy gradients were retained separately for descriptive interpretation but were not incorporated into the trait-based similarity network.

Variable	Description	Reason excluded from incidence matrix construction
forewing_minimum	Minimum forewing length (mm)	Continuous morphology variable outside categorical strategy encoding framework
forewing_maximum	Maximum forewing length (mm)	
estimated_dry_mass	Estimated adult dry mass (mg)	
gb_10_km_squares_(2000–2016)	Number of occupied 10 km grid squares in Great Britain	Monitoring-derived distribution metric reflecting sampling coverage rather than ecological strategy
ireland_10_km_squares_(2000–2016)	Number of occupied 10 km grid squares in Ireland	
long_term_distribution_trend_gb	Long-term distribution change (%)	Monitoring-derived population trend not used in trait-based similarity construction
short_term_distribution_trend_gb	Short-term distribution change (%)	
abundance_trend	Long-term abundance change (%)	
phenology_shift	Change in mean flight timing (days) between historical periods	Derived monitoring-based phenological change metric rather than categorical life-history state
light_value	Ellenberg indicator value for hostplant light preference	Hostplant environmental proxy indicator rather than species-level ecological trait state
moisture_value	Ellenberg indicator value for hostplant moisture preference	
reaction	Ellenberg indicator value for soil reaction (pH proxy)	
nitrogen	Ellenberg indicator value for nitrogen availability	
salt_tolerance	Ellenberg indicator value for salinity tolerance	

Prior to network construction, the incidence matrix was subjected to a preliminary filtering step to remove uninformative and redundant trait-value columns. Trait-values present in fewer than five species or in more than 99% of species were excluded, as such features provide little discriminatory power for similarity-based analyses. To remove highly similar trait categories, pairwise Jaccard similarity was computed between all remaining binary trait-value columns, and one column was removed from pairs with similarity greater than 0.95. When redundant columns were identified, retention was based on semantic clarity and data quality, with confirmed states preferred over uncertain or provisional encodings. This procedure reduced dimensionality while preserving a fully interpretable, binary encoded representation of ecological trait states used for similarity computation. A complete list of removed redundant trait-values and their retained representatives is provided in Supplementary S1 Table.

### Conservation status for post hoc interpretation

Conservation status was not used in network construction, feature selection, or community detection. Instead, regional Red List assessments were examined post hoc to interpret differences in conservation risk across the ecologically defined communities. Because conservation status was incorporated only after community detection was completed, all reported associations between community membership and extinction risk reflect post hoc comparisons and were not part of the clustering procedure.

Species-level conservation status was obtained from independent Red List assessments for Great Britain and Ireland, which reflect region-specific population trends, habitat availability, and conservation pressures. When Red List categories were collapsed into a binary threatened versus not threatened classification, the two assessments were the same for approximately 70% of species with data available in both regions, indicating substantial, though incomplete, agreement between national assessments.

For post hoc summaries and statistical analyses, species classified as Vulnerable (VU), Endangered (EN), or Critically Endangered (CR) were labeled as *threatened*, while species classified as Least Concern (LC) or Near Threatened (NT) were labeled as *not threatened*. Species listed as Data Deficient (DD), Not Evaluated (NE), or Regionally Extinct (RE) were excluded from proportional summaries, as these categories do not represent confirmed low-risk states. The resulting distribution of species across categories is summarized in Table 2.

**Table 2:** Summary of binary conservation status targets derived from regional Red List assessments for Great Britain and Ireland.

Region	Threatened (1)	Not threatened (0)	Excluded (NA)
Great Britain	71	724	173
Ireland	49	464	455

Great Britain conservation status was used as the primary reference due to its more complete coverage within the dataset. As a consistency check, community-level summaries were also computed using Ireland Red List classifications for species with available data. Similar patterns across regions suggest that the observed associations between community structure and conservation risk were not driven by differences between national assessments.

### Network-aware feature selection via bootstrap stability

Following trait encoding and preliminary filtering, we applied a stability-based feature selection procedure to identify trait families that contributed meaningfully to species similarity structure. Trait families were defined as groups of related binary trait-value encodings derived from the same underlying ecological attribute (for example, monthly indicators describing egg phenology, alternative pupation environments, or categories of larval host association). A complete listing of trait families and their associated encoded variables is provided in S1 Table, and a summary of trait families with examples is included in Supplemental S2 Table. Feature selection was conducted at the level of trait families rather than individual trait-values in order to preserve ecological interpretability and avoid ad hoc exclusion of closely related encodings.

Trait families were evaluated using a bootstrap-based stability framework in which their contribution to clustering consistency was assessed by measuring changes in adjusted Rand index (ARI) following systematic removal. This approach was conceptually informed by prior work on stability selection and clustering validation, in which reproducibility of structure under resampling is used as a criterion for identifying informative features [23,39,40].

Using a fixed network construction and clustering procedure, we first quantified baseline clustering stability relative to the clustering obtained from the full incidence matrix through bootstrap resampling of species. In each bootstrap replicate, a subset of species was resampled with replacement, the species similarity network was reconstructed using the same similarity metric and neighborhood parameter settings, and community detection was re-applied. Stability was quantified by comparing community assignments in each bootstrap replicate to the baseline clustering using the adjusted Rand index (ARI).

Trait families were then evaluated by ablation. For each family, all associated trait-values were removed from the incidence matrix and the bootstrap stability analysis was repeated under the same network construction and clustering settings. Trait families whose removal did not reduce clustering stability beyond a predefined threshold (ΔARI ≤ −0.02), chosen to represent a small but meaningful reduction in clustering agreement under bootstrap resampling, were excluded from the final incidence matrix, whereas families whose removal produced a larger reduction in stability were retained. The resulting reduced trait set was fixed prior to all downstream network construction, clustering, and interpretation to avoid circularity between feature selection and conservation-status analysis.

### Network construction and community detection

Species similarity networks were constructed from the final reduced trait incidence matrix obtained through network-aware feature selection. Pairwise similarity between species was computed using the Jaccard similarity coefficient, which measures the proportion of shared trait indicators relative to the total number present in either species. This presence–absence–based measure is appropriate for sparse, categorical trait data because it ignores cases where both species lack a trait.

Similarity values were used to construct a weighted, undirected species–species network using a union *k*-nearest-neighbor (kNN) approach. For each species, edges were retained to its *k* most similar neighbors, and the union of these neighbor sets was used to define the network, such that an edge was included if either species ranked the other among its *k* nearest neighbors. Edge weights corresponded to pairwise similarity values. Following evaluation of network robustness across a range of *k* values (see below), a single value of *k* was fixed for all final analyses. Self-loops were excluded, and multiple edges between species pairs were collapsed by retaining the maximum observed similarity weight.

Community structure in the resulting species similarity network was identified using the Leiden community detection algorithm [25], which optimizes modularity and yields well-connected communities in weighted networks. Applied to the weighted network with fixed parameters, the algorithm produced a baseline partition that served as the basis for all subsequent analyses and interpretation. The number and composition of communities emerged from the data through the algorithmic procedure and were not imposed a priori.

As a robustness check, we also applied the Louvain algorithm [41] to the same network and compared the resulting partition to the Leiden solution using the adjusted Rand index (ARI). The two methods produced highly similar community assignments (ARI = 0.886), and the Leiden partition was used as the primary clustering for all reported results.

### Selection of neighborhood size (*k*)

The choice of *k*, or neighborhood size, in *k*-nearest-neighbor (kNN) graph construction influences network connectivity and the stability of detected communities. To evaluate sensitivity to this parameter, we assessed a range of candidate values of *k* prior to final network construction.

Using the final reduced trait incidence matrix, weighted species similarity networks were constructed for k∈{5,8,10,12,15,20,25} using a union-*k*NN approach. For each value of *k*, we computed basic graph properties, including the number of connected components, the fraction of species in the largest connected component, and average node degree. Community stability was evaluated using the bootstrap resampling procedure described above. For each *k*, a baseline clustering was obtained using the Leiden algorithm, and bootstrap-derived clusterings were compared to this baseline using the ARI.

Across the tested values of *k*, networks were predominantly connected, with more than 99% of species contained in the largest connected component. Community stability, as measured by mean ARI across bootstrap replicates, was maximized at *k* = 10. Based on this analysis, we selected *k* = 10 as the smallest neighborhood size yielding a stable and well-connected network and used this value for all subsequent analyses (Table 3).

**Table 3:** Sensitivity analysis for selection of neighborhood size (*k*) in species similarity network construction. For each value of *k*, we report basic graph properties and bootstrap community stability measured by the ARI. The final value (*k* = 10) was selected as the smallest neighborhood size yielding a predominantly connected network and maximal stability.

*k*	Edges	Components	Largest component fraction	Avg. degree	Mean ARI	SD ARI
5	3,540	2	0.995	7.31	0.619	0.049
8	5,568	2	0.995	11.50	0.707	0.044
**10**	**6,877**	**2**	**0.995**	**14.20**	**0.812**	**0.062**
12	8,155	2	0.995	16.85	0.778	0.052
15	10,145	2	0.995	20.96	0.787	0.052
20	13,431	2	0.995	27.75	0.804	0.054
25	16,642	2	0.995	34.38	0.779	0.049

### Software and computational environment

All analyses were performed using custom Python scripts implementing the trait similarity network construction, stability analysis, and community detection workflow described above. The analysis pipeline used standard scientific computing libraries including NumPy, pandas, scikit-learn, and NetworkX, together with the Leiden community detection implementation available through the leidenalg package.

## Results

### Global structure of the Lepidoptera trait similarity network

The final species similarity network comprised 968 Lepidoptera (73 butterfly species and 895 moth species) connected by 6,877 weighted edges, with more than 99% of species contained within a single connected component. Edge weights reflected pairwise ecological similarity based on Jaccard overlap of trait profiles, producing a heterogeneous distribution of similarity strengths across the network. Bootstrap analyses indicated that community assignments were stable across a broad range of neighborhood sizes (*k* = 8–25), with maximal stability observed at *k* = 10. This value was therefore used for all subsequent analyses.

**Fig 2:**
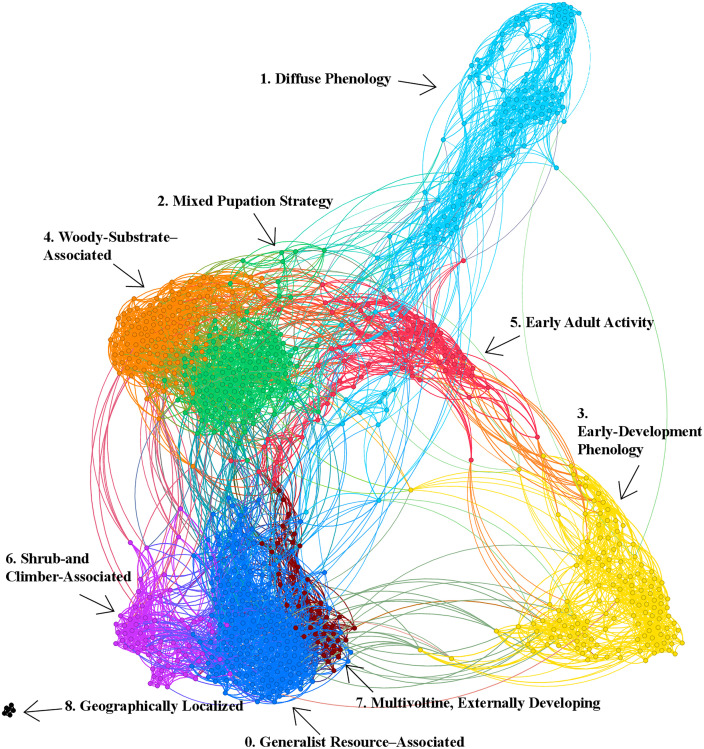
Species similarity network constructed from Lepidoptera trait data. Nodes represent species and edges represent weighted trait similarity (Jaccard-based, union-*k*NN with *k* = 10). Node colors indicate Leiden communities, revealing clear modular structure with nine detected groups. Communities are described in Table 4.

Application of the Leiden community detection algorithm to the weighted, union-*k*NN network identified clear modular structure (Fig 2). Nine communities were detected, with community sizes ranging from 5 to 208 species (Table 4). Most communities were dominated numerically by macro-moths, whereas Community 7 differed in containing a majority of butterflies (58.7%). No additional small satellite communities were observed beyond these nine partitions. The resulting partition exhibited dense intra-community connectivity and comparatively sparse inter-community connections. Conservation status was not used in network construction or clustering, allowing conservation outcomes to be interpreted as post hoc associations with network-derived ecological structure, not as inputs to the clustering procedure.

**Table 4:** Summary of Leiden network communities derived from the species similarity network. For each community, the number of species and the number (and percentage) classified as threatened are shown for Great Britain (GB) and Ireland (IE), with some counted in both regional summaries because they are classified as threatened in both GB and IE. Ecological profiles are summarized using the most common traits per community, based on prevalence relative to the full species pool.

Community	*n* species	*n* (%) butterfly	*n* (%) moth	GB threatened	IE threatened	Dominant trait–value indicators
0 – Generalist resource–associated	208	2 (1%)	206 (99%)	12 (5.8%)	12 (5.8%)	Larval resources include lichens and algae; Broad host associations; Generalist feeding indicators; Mixed developmental substrates; Widespread habitat use
1 – Diffuse phenology	146	14 (10%)	132 (90%)	0 (0.0%)	0 (0.0%)	Uncertain or flexible larval timing across winter months; Broad seasonal distribution of larval timing; Weak specialization in developmental traits
2 – Mixed pupation strategies	145	7 (5%)	138 (95%)	10 (6.9%)	8 (5.5%)	Multiple pupation strategies; Mixed developmental timing and pupation patterns; Mixed host plant categories; Flexible developmental contexts
3 – Early-development phenology	140	9 (6%)	131 (94%)	23 (16.4%)	11 (7.9%)	Early-season egg presence; Extended egg phenology into autumn and winter; Constrained early developmental timing
4 – Woody-substrate–associated	136	1 (1%)	135 (99%)	6 (4.4%)	4 (2.9%)	Associations with woody substrates and decaying plant material; Tree-linked larval environments; Development associated with structurally complex woody substrates
5 – Early adult activity	75	11 (15%)	64 (85%)	2 (2.7%)	2 (2.7%)	Early adult activity; Winter or very early spring flight periods; Broad habitat tolerance; Adult-stage phenological emphasis
6 – Shrub- and climber-associated	67	2 (3%)	65 (97%)	3 (4.5%)	2 (3.0%)	Larval development on shrubs and climbers; Multiple larval stages within a season; Development associated with shrubs, climbers, and related plant structures
7 – Multivoltine, externally developing	46	27 (59%)	19 (41%)	15 (32.6%)	10 (21.7%)	Multiple larval generations per year; External vegetation use during development; Narrow developmental windows; Repeated seasonal developmental cycles
8 – Geographically localized	5	0 (0%)	5 (100%)	0 (0.0%)	0 (0.0%)	Strong geographic localization signals; Restricted regional occurrence; Limited ecological diversity within a small community

For reference, communities are assigned numeric identifiers (0–8). Descriptive labels based on dominant trait–value prevalence are provided solely for interpretive convenience and are not used in statistical analyses. These labels include generalist resource–associated (Community 0), diffuse phenology (Community 1), mixed pupation strategies (Community 2), early-development phenology (Community 3), woody-substrate–associated (Community 4), early adult activity (Community 5), shrub- and climber-associated (Community 6), multivoltine, externally developing (Community 7), and geographically localized species (Community 8).

Table 4 summarizes community sizes, conservation status distributions for Great Britain and Ireland, and the most prevalent trait–value indicators within each community relative to the full species pool.

### Statistical validation of community-level conservation patterns

To assess whether threatened species were non-randomly distributed across ecological communities, we tested for independence between Leiden community membership and threatened status in Great Britain. Threatened species were unevenly distributed across communities ($\chi^{2}(7)=54.40$, *p* = 1.96 × 10^−9^), indicating a significant association between community membership and conservation status. Observed and expected counts underlying the global chi-square test are reported in S3 Table (Supporting Information). Community 8 was excluded from community-level statistical testing because its small size was below the threshold required for valid chi-square and Fisher’s exact tests.

Community-level enrichment tests were performed using Fisher’s exact test as an exact conditional test of association in 2×2 contingency tables. Although originally derived under fixed-margin assumptions, Fisher’s test is widely used for testing independence in observational contingency tables with small expected counts [42]. Community 7 exhibited the largest odds ratio (5.99, *p* = 2.0 × 10^−6^), indicating a substantially higher prevalence of threatened species relative to the remainder of the network. Community 3 also showed a significantly elevated odds ratio (2.62, *p* = 7.6 × 10^−4^). Several communities exhibited lower odds ratios or no statistically detectable deviation from the network-wide baseline. Results are summarized in Table 5.

**Table 5:** Community-level associations between Leiden network communities and threatened status in Great Britain. Odds ratios compare each community against all others combined. Community 8 is omitted due to insufficient sample size for contingency-based statistical testing.

Community	Odds ratio	Fisher’s exact *p*
0 – Generalist resource–associated	0.57	0.088
1 – Diffuse phenology	0.00	0.612
2 – Mixed pupation strategies	0.85	0.737
3 – Early-development phenology	2.62	7.6 × 10^−4^
4 – Woody-substrate–associated	0.43	0.047
5 – Early adult activity	0.27	0.052
6 – Shrub- and climber-associated	0.45	0.261
7 – Multivoltine, externally developing	**5.99**	2.0 × 10^−6^

To evaluate whether detected communities corresponded to differences in trait composition, permutational multivariate analysis of variance (PERMANOVA) was applied to the Jaccard distance matrix of species trait profiles. Trait composition differed significantly among communities (PERMANOVA on Jaccard distances: pseudo-*F* = 66.08, df = 8, *R*^2^ = 0.40, 999 permutations, *p* = 0.001).

### Ecological differentiation of network communities

Phenological traits varied strongly across network communities. Communities 3 and 5 were characterized by early-season life stages, including early adult activity and early egg or larval presence, whereas Community 1 exhibited diffuse phenological signals spanning extended portions of the annual cycle. In contrast, other communities showed more temporally constrained activity windows, with life stages concentrated within narrower seasonal periods (Table 4).

Life-cycle attributes further differentiated communities, particularly with respect to voltinism and developmental context. Community 7 showed a high prevalence of multivoltine species with multiple generations per year, whereas several larger communities were dominated by univoltine species with more tightly constrained seasonal developmental schedules. Communities also differed in dominant pupation environments and developmental substrates, including contrasts between woody-associated development, externally exposed development, and mixed-substrate life histories.

Across communities, trait differentiation was not driven by any single ecological dimension. Instead, community membership reflected consistent combinations of phenological timing, voltinism, and developmental traits, indicating that network communities captured recurring patterns of trait co-occurrence across multiple ecological axes. This pattern is consistent with the interpretation of network communities as integrated ecological strategy groups defined by multivariate trait structure.

### Network position and conservation vulnerability

The distribution of conservation status varied across ecologically defined communities (Table 4). Several communities exhibited low proportions of threatened species across both Great Britain and Ireland, whereas others showed elevated threat prevalence. Community 7 contained the highest proportion of threatened species, with 32.6% classified as threatened in Great Britain and 21.7% in Ireland, despite representing a relatively small fraction of the overall network.

Although Community 7 exhibited the highest proportion of threatened species, Community 3 contained the largest absolute number of threatened species. This pattern reflects the larger size of Community 3 combined with a moderately elevated threat prevalence relative to most other communities (Table 4). In contrast, Community 7 combined high threat prevalence with comparatively small community size.

**Fig 3:**
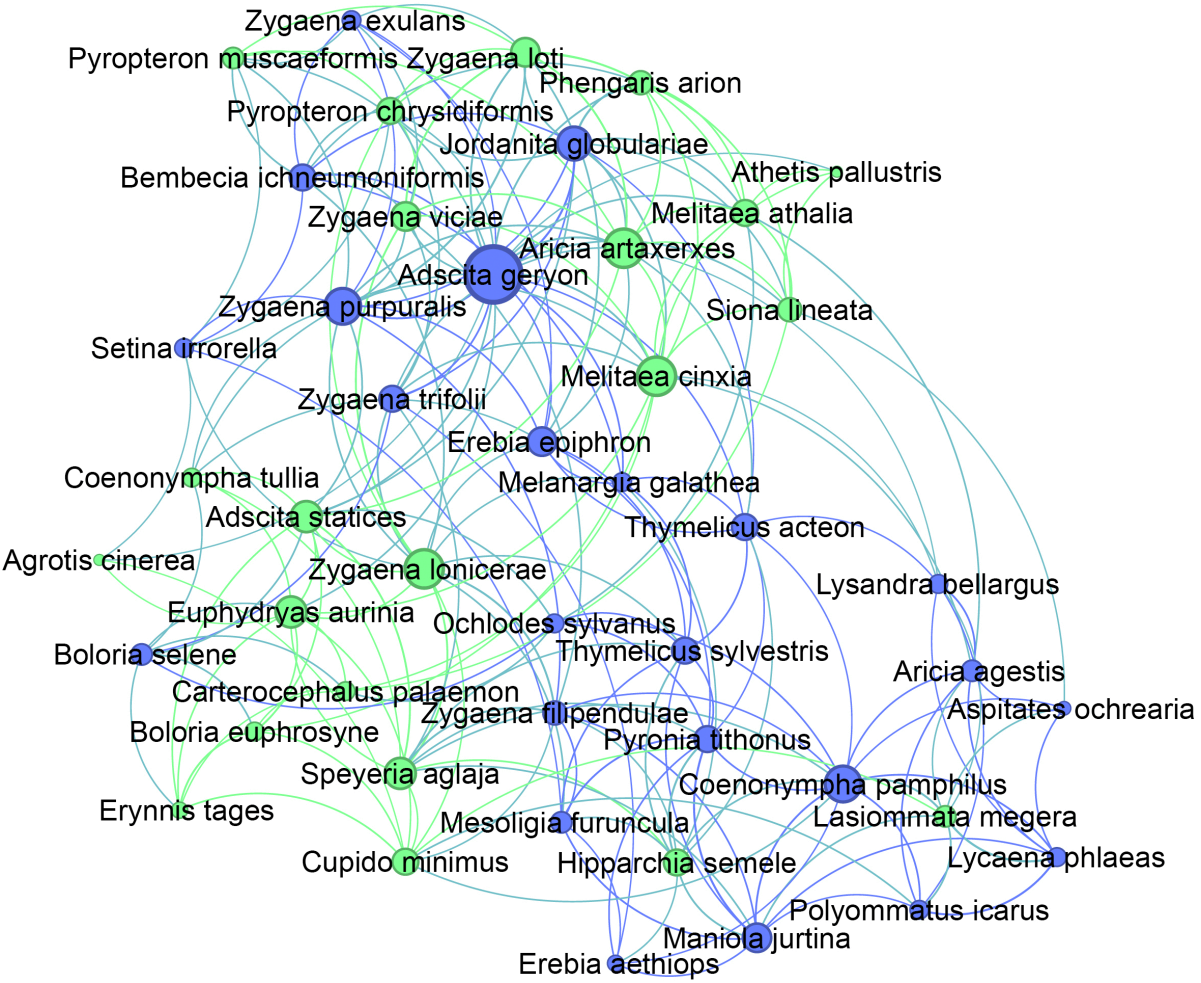
Subnetwork corresponding to Community 7 (multivoltine, externally developing species). Nodes represent individual species within Community 7 and edges represent weighted trait similarity among species in the community. Node size indicates degree. Green nodes are threatened.

The subnetwork corresponding to Community 7 formed a densely connected region of the global trait similarity network (Fig 3). Within this subnetwork, threatened species were distributed throughout the community rather than confined to peripheral positions. Node degree varied across species, with several highly connected nodes also classified as threatened. Among the five species with the highest degree within Community 7, three were classified as threatened: *Melitaea cinxia* (Endangered in Great Britain, degree = 15), *Aricia artaxerxes* (Vulnerable in Great Britain, degree = 15), and *Zygaena lonicerae* (Vulnerable in Ireland, degree = 15). The most highly connected species, *Adscita geryon*, had a degree of 22. Across the community as a whole, Community 7 contained 46 species, of which 15 (32.6%) were classified as threatened in Great Britain and 10 (21.7%) in Ireland, including species spanning a range of degree values (e.g., *Coenonympha tullia*, degree = 7; *Euphydryas aurinia*, degree = 12; *Phengaris arion*, degree = 9; *Hipparchia semele*, degree = 10; *Speyeria aglaja*, degree = 12).

**Fig 4:**
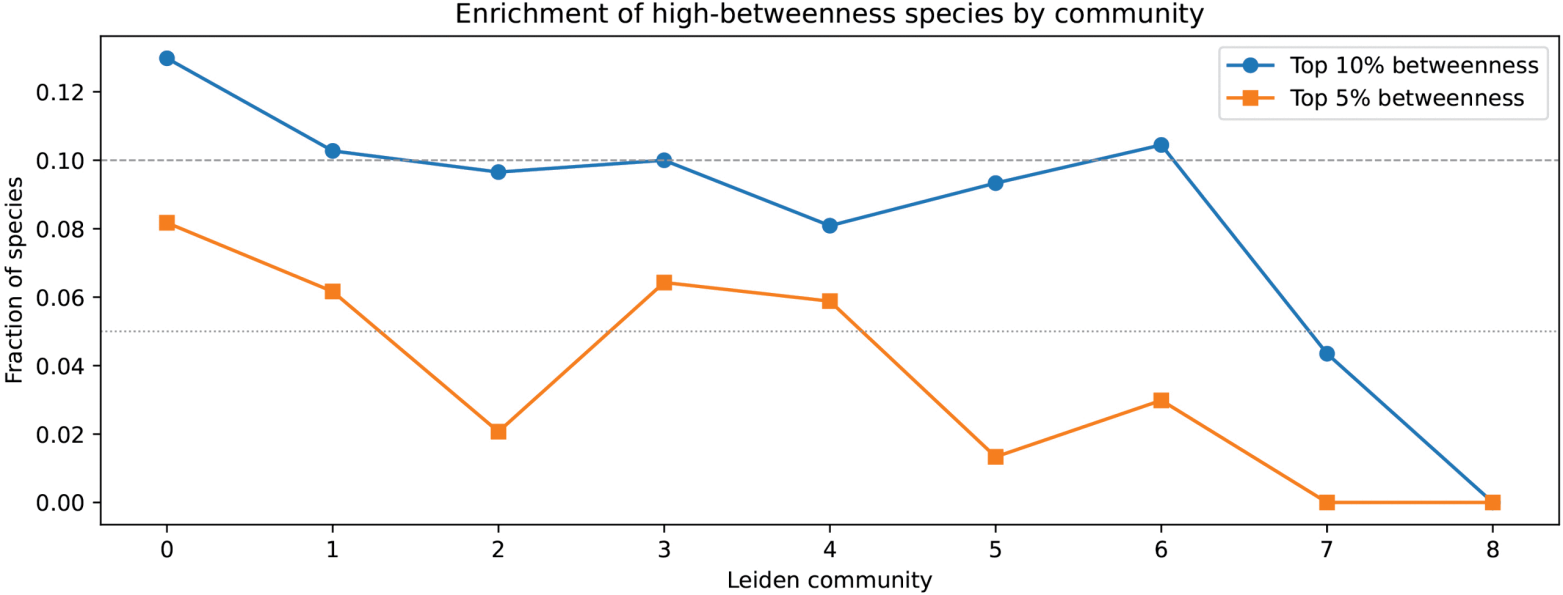
Enrichment of high-betweenness species by community. For each Leiden community, the fraction of species falling within the top 5% and top 10% of betweenness centrality values is shown. Horizontal dashed lines indicate the expected fractions under a uniform distribution.

Betweenness centrality was examined to assess whether extinction risk was associated with structurally central positions in trait similarity space or instead concentrated within particular ecological strategies. Species in Community 7 were underrepresented among high-betweenness nodes (Fig 4). This indicates that extinction vulnerability in this system is not associated with occupying structurally central or bridging positions within trait similarity space. No species from this community fell in the top 5% of betweenness values, and only a small number appeared in the top 10%. In contrast, several communities with lower threat prevalence contained a disproportionately larger share of high-betweenness species, indicating that structural centrality alone does not explain the observed distribution of extinction risk across the network.

## Discussion

This study demonstrates that ecological similarity among Lepidoptera is structured by integrated combinations of phenological and developmental traits, and that extinction risk is unevenly distributed across these trait-defined strategies. The goal of this analysis is not to identify single traits that predict extinction risk, but to test whether vulnerability concentrates within combinations of traits defining ecological strategies. By extending a trait similarity network framework from birds to Lepidoptera, our results show that clustered conservation vulnerability can emerge in taxa with fundamentally different life history structures. Below, we interpret these patterns in terms of ecological strategy, network structure, and their implications for understanding conservation vulnerability.

### Network communities as integrated ecological strategies

The emergence of discrete communities in the trait similarity network suggests that ecological similarity among Lepidoptera is organized into a limited number of clearly defined groups in trait space. This structure does not resemble a continuous spectrum of partially overlapping trait combinations, but instead reflects recurring multivariate trait assemblages. Such clustering aligns with the concept of ecological syndromes, in which sets of traits co-occur because they reflect shared life-history strategies [43]. More broadly, these patterns are consistent with work on life-history trade-offs, which emphasizes that traits covary due to underlying constraints and are not expected to vary independently [44].

From a conservation perspective, this pattern has important implications. When species cluster into discrete ecological strategy groups, vulnerability to environmental change may be shared among members of the same group, even when no single trait is rare or extreme. In networked ecological systems, such modular organization often corresponds to functionally cohesive groups that respond in similar ways to environmental disturbance and therefore share similar sensitivities to environmental change [45].

The communities in this study were identified using an unsupervised approach that did not incorporate conservation status. The association between community membership and extinction risk therefore arises from how traits naturally group together and not from circular classification. This supports the interpretation of network communities as biologically meaningful units for examining conservation vulnerability at the level of integrated ecological strategies.

The ecological dimensions underlying this integration were dominated by phenological timing, referring to when during the year different life stages occur, and by developmental context, while habitat and trophic specialization played a more limited role. Communities differed in the timing and duration of adult activity, the seasonal scheduling of immature stages, and key aspects of life-cycle structure, including voltinism and pupation environment. Together, these patterns underscore the importance of seasonal timing and developmental exposure in shaping Lepidoptera ecological strategies, helping to explain why trait combinations related to phenology and development emerged as primary organizers of community structure.

### Generality of trait similarity networks across taxa

The application of trait similarity networks to both birds and Lepidoptera suggests that this framework captures general features of ecological organization that are not specific to any single taxonomic group. In both systems, unsupervised network analysis revealed a limited number of coherent communities defined by combinations of traits, with conservation vulnerability concentrated within particular strategy groups instead of being distributed evenly across species [12].

At the same time, the ecological dimensions organizing these communities differed in biologically meaningful ways. In birds, communities were structured primarily by traits related to foraging, movement, and habitat use, whereas in Lepidoptera, phenological and developmental characteristics played a dominant role. This contrast is consistent with fundamental differences in life-history constraints between taxa and does not appear to reflect limitations of the analytical framework itself [46].

### Why Community 7 is associated with elevated extinction risk

Among the nine network communities, Community 7 exhibited the highest per-species prevalence of threatened status across both Great Britain and Ireland, despite representing a relatively small fraction of the overall network. In contrast, Community 3 contained a larger absolute number of threatened species due to its greater size combined with moderately elevated risk. This distinction highlights the difference between concentrated vulnerability within a narrowly defined ecological strategy and broader conservation burden distributed across a more prevalent strategy group [47].

The elevated vulnerability observed in Community 7 appears to reflect the combined ecological strategy represented by this group, not any single component in isolation. In particular, risk may arise from the combination of multiple generations per year, externally exposed development, and relatively narrow developmental windows. Multiple generations per year may increase cumulative exposure to seasonal habitat disturbance, such as pesticide application timing or mowing schedules, while external vegetation use during development constrains larval foraging to available host plants and limits flexibility under habitat loss or fragmentation. In addition, narrow developmental windows may reduce the capacity of species to track phenological shifts in host plant availability under climate change, leading to shared sensitivities across species occupying this strategy. Together, these features suggest that vulnerability emerges from the interaction among phenological timing, developmental exposure, and life-cycle turnover, not from any single ecological attribute alone.

Network structure provides additional context for interpreting this pattern. Species within Community 7 formed a tightly connected and internally cohesive cluster, but were weakly connected to other regions of the trait similarity network and underrepresented among high-betweenness nodes. This indicates that elevated vulnerability appears to be associated with ecological isolation within trait space, not structural centrality in the global network. Such compartmentalization has been shown to reflect meaningful ecological structure and to influence system responses to environmental change, with relatively isolated modules often exhibiting heightened sensitivity because limited connectivity may reduce the availability of alternative ecological strategies under environmental change [48]. In this system, elevated extinction risk was associated with ecological isolation in trait space and not with globally central or bridging positions, emphasizing the role of ecological strategy configuration in shaping vulnerability [36].

Importantly, elevated vulnerability in Community 7 was not confined to a small number of marginal species. Highly connected species within this community were frequently classified as threatened, indicating that risk is embedded within the core of the strategy rather than arising from peripheral or anomalous cases. This pattern raises the possibility that declines affecting central species could have broader consequences for the persistence of this ecological strategy as a whole, particularly in systems where ecological responses to environmental change are tightly coupled to shared life-history constraints.

### Implications for monitoring and conservation

The network-based framework presented here has implications for how conservation vulnerability is monitored and interpreted. By identifying groups of species that share ecological strategy profiles, trait similarity networks enable a shift from single-species or single-trait assessments toward a strategy-level perspective that allows conservation monitoring to identify groups of species likely to share sensitivities to habitat change, climate shifts, or other disturbances. Because ecological strategies reflect shared life-history constraints, declines affecting one species within a vulnerable strategy group may signal emerging risk for others occupying similar regions of ecological trait space. This perspective is particularly relevant in systems where vulnerability reflects the joint configuration of multiple ecological traits.

From a monitoring standpoint, communities defined by shared ecological strategies provide a useful unit for assessing coordinated responses to environmental change. Monitoring could focus on representative species within strategy groups that exhibit elevated vulnerability, with changes in their status serving as early indicators of broader strategy-level decline. This approach complements existing species-based monitoring programs by highlighting patterns of shared sensitivity that may be overlooked when species are assessed individually [49].

More broadly, the network perspective suggests that conservation prioritization may benefit from considering how ecological strategies are distributed within trait space. Strategies that are both specialized and weakly connected to alternative strategies may warrant particular attention from a precautionary conservation perspective [50], even when the species within them are not currently classified as highly threatened. By emphasizing the configuration of ecological strategies in addition to their prevalence, trait similarity networks provide a flexible framework for integrating trait-based data into conservation decision-making without requiring taxon-specific predictive models.

Taken together, these implications highlight the potential of network-based trait analyses to assist conservation planning by identifying vulnerable regions of ecological strategy space. While further work is needed to link such patterns directly to management outcomes, this approach offers a scalable and interpretable way to incorporate multidimensional ecological information into assessments of conservation vulnerability at the level of integrated ecological strategies. In this context, species belonging to Community 7 may warrant particular attention in future conservation review and assessment efforts, as their shared ecological strategy was associated with consistently elevated threat prevalence across both regional Red List datasets examined here.

### Generality and future directions

While the present study focuses on static representations of ecological similarity, the network-based framework naturally lends itself to several extensions. Trait similarity networks could be expanded to incorporate temporal dynamics, allowing changes in species’ positions within ecological trait space to be tracked as environmental conditions shift. Such approaches may be particularly informative in systems experiencing rapid phenological or life-history change.

Future work could also integrate additional data layers, including abundance, geographic overlap, or phylogenetic relationships, using more complex network models. These extensions would enable ecological similarity to be examined alongside spatial or evolutionary structure, providing a richer view of how vulnerability emerges across interacting dimensions.

An important next step would be to integrate long-term monitoring data, such as population trend indices from the UK Butterfly Monitoring Scheme, to evaluate whether communities identified in trait similarity space differ systematically in observed rates of decline. Such integration would provide a valuable test of whether strategy-level vulnerability inferred from trait structure corresponds directly to observed contemporary population trajectories.

Finally, the stability-aware feature selection and network construction pipeline developed here is not restricted to Lepidoptera or to the specific trait sets analyzed in this study. As trait databases continue to expand across taxa, network-based approaches provide a scalable and interpretable means of exploring multidimensional ecological structure, supporting comparative analyses of conservation vulnerability across diverse taxa and ecological systems.

## Conclusion

In this study, we applied a trait similarity network framework to examine how ecological strategies structure extinction risk in Lepidoptera, extending an approach previously applied to seabirds. By constructing a species similarity network from a set of ecological traits and identifying communities using unsupervised network analysis, we showed that butterfly and macro-moth species cluster into a limited number of integrated ecological strategies defined primarily by phenological timing and developmental context. Conservation status, introduced only post hoc, was unevenly distributed across these strategy groups, revealing concentrated vulnerability that would not be apparent from single-trait or single-species analyses alone.

Our results demonstrate that extinction risk in Lepidoptera is organized at the level of ecological strategies rather than being randomly distributed across species. In particular, the identification of a small, ecologically cohesive community with elevated per-species threat prevalence highlights how network-based representations of trait similarity can identify regions of ecological strategy space associated with heightened vulnerability and deserving further conservation attention. At the same time, contrasts between per-species risk and absolute conservation burden across communities underscore the importance of considering both dimensions when interpreting conservation patterns and prioritizing monitoring efforts.

More broadly, the consistency of strategy-level structure observed here and in previous seabird analyses suggests that trait similarity networks provide a general and scalable framework for linking multidimensional ecological traits to conservation outcomes across taxa with fundamentally different life histories. By focusing on ecological strategies as multivariate configurations within trait space, this approach complements existing conservation assessments and supports strategy-level monitoring frameworks that may help identify emerging risk in data-limited systems such as insects. As trait databases and long-term monitoring resources continue to expand, integrating trait similarity networks with population trend data offers a promising path toward more interpretable and comparative assessments of conservation vulnerability across ecological systems.

## Supporting information

S1 Table Complete listing of trait families and encoded trait-value variables included in the species–trait incidence matrix prior to stability-based feature selection. Trait families correspond to groups of related binary encodings derived from a shared ecological attribute (for example, monthly indicators describing egg phenology, alternative pupation environments, or categories of larval host association). Each row lists an individual encoded trait-value together with its corresponding trait family and description as defined in the trait dictionary used to construct the incidence matrix. This table documents the full structure of the encoded trait dataset used as input to the bootstrap stability feature-selection procedure and indicates which families were retained in the reduced trait set used for downstream network construction. Variable descriptions follow the definitions provided in the original NERC Environmental Information Data Centre trait documentation.
(CSV)

S2 Table Summary of trait families included in the Lepidoptera species–trait incidence matrix before and after filtering. This table lists the ecological trait families used to construct the species–trait incidence matrix, along with example encoded variables and the number of trait-value columns initially available in each family and retained after preprocessing. Filtering steps included removal of rare or near-universal trait-values based on prevalence thresholds and elimination of redundant variables using pairwise similarity criteria. The retained trait families formed the basis for subsequent network construction and community detection analyses.
(CSV)

S3 Table Expected counts underlying the chi-square test. Table S3 reports the observed and expected counts of threatened and non-threatened species within each Leiden community under the null hypothesis that conservation status is independent of community membership (Great Britain Red List status). Expected values were derived from the chi-square test of independence described in the Results. This table provides the contingency structure underlying the global chi-square statistic and highlights which communities contribute most strongly to deviations from random expectation. Community 8 is omitted due to its small size, which results in expected counts below the threshold required for chi-square testing.
(CSV)

S4 Table Taxonomic designation of Lepidoptera species as butterflies or macro-moths. This table provides the classification of each species included in the analysis as either a butterfly or a macro-moth, based on standard usage in British and Irish Lepidoptera recording and ecological literature. These designations were used for descriptive summaries and supplemental comparisons only and were not incorporated into trait encoding, feature selection, network construction, or community detection analyses. The table supports interpretation of results that distinguish patterns between butterflies and macro-moths across the detected trait-based communities.
(CSV)
